# Cardiovascular Benefits of Native GLP-1 and its Metabolites: An Indicator for GLP-1-Therapy Strategies

**DOI:** 10.3389/fphys.2017.00015

**Published:** 2017-01-30

**Authors:** Junfeng Li, Juan Zheng, Susanne Wang, Harry K. Lau, Ali Fathi, Qinghua Wang

**Affiliations:** ^1^Department of Endocrinology and Metabolism, Huashan Hospital, Shanghai Medical College, Fudan UniversityShanghai, China; ^2^Department of Endocrinology, Renmin Hospital of Wuhan UniversityWuhan, China; ^3^Division of Endocrinology and Metabolism, the Keenan Research Centre in the Li Ka Shing Knowledge Institute, St. Michael's HospitalToronto, ON, Canada; ^4^Department of Physiology, Faculty of Medicine, University of TorontoToronto, ON, Canada

**Keywords:** GLP-1, T2DM, DPP4, cardiovascular diseases, cardiac protection

## Abstract

Cardiovascular disease is a common co-morbidity and leading cause of death in patients with type 2 diabetes mellitus (T2DM). Glucagon-like peptide 1 (GLP-1) is a peptide hormone produced by intestinal L cells in response to feeding. Native GLP-1 (7-36) amide is rapidly degraded by diaminopeptidyl peptidase-4 (DPP4) to GLP-1 (9-36) amide, making 9-36a the major circulating form. While it is 7-36a, and not its metabolites, which exerts trophic effects on islet β-cells, recent studies suggest that both 7-36a and its metabolites have direct cardiovascular effects, including preserving cardiomyocyte viability, ameliorating cardiac function, and vasodilation. In particular, the difference in cardiovascular effects between 7-36a and 9-36a is attracting attention. Growing evidence has strengthened the presumption that their cardiovascular effects are overlapping, but distinct and complementary to each other; 7-36a exerts cardiovascular effects in a GLP-1 receptor (GLP-1R) dependent pathway, whereas 9-36a does so in a GLP-1R independent pathway. GLP-1 therapies have been developed using two main strategies: DPP4-resistant GLP-1 analogs/GLP-1R agonists and DPP4 inhibitors, which both aim to prolong the life-time of circulating 7-36a. One prominent concern that should be addressed is that the cardiovascular benefits of 9-36a are lacking in these strategies. This review attempts to differentiate the cardiovascular effects between 7-36a and 9-36a in order to provide new insights into GLP-1 physiology, and facilitate our efforts to develop a superior GLP-1-therapy strategy for T2DM and cardiovascular diseases.

## Introduction

Cardiovascular complications are the primary co-morbidity and leading cause of death in patients with type 2 diabetes mellitus (T2DM; Killilea, 2002; Mazzone et al., 2008). Epidemiological studies have shown that patients with cardiovascular disease benefit from good glycemic and lipid control (AM Committee, 2001; Balkau et al., 2004). Besides its indirect cardiac actions through the control of glucose and lipid metabolism by modulating insulin and glucagon secretion, GLP-1 may exert direct effects on heart and blood vessels (Ravassa et al., 2012; Ussher and Drucker, 2012).

GLP-1 is produced in response to meal ingestion in intestinal L cells, where it is synthesized as part of the proglucagon polypeptide, and released following tissue specific post-translational processing (Drucker, 2006; Brubaker, 2010). The biological functions of GLP-1 include stimulating insulin secretion in a glucose-concentration-dependent fashion, suppressing glucagon release, promoting satiety, and increasing peripheral glucose disposal, all of which render this incretin to be an attractive therapeutic target for T2DM (Baggio and Drucker, 2007; Brubaker, 2010; Donnelly, 2012).

**Figure 1:**
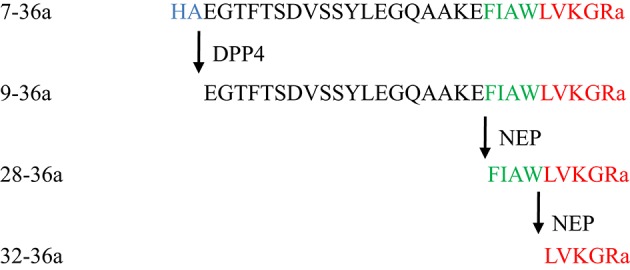
**Structures of native GLP-1 and its metabolites**.

Native GLP-1, 7-36a, has a short half-life (1–2 min) due to rapid enzymatic degradation by DPP4 as well as other enzymes (Hupe-Sodmann et al., 1995). The DPP4-cleaved form of GLP-1, 9-36a, is the predominant circulating form (Tomas and Habener, 2010) (Figure 1). Since 9-36a has no significant insulin-stimulatory effects and weak affinity to the GLP-1 receptor (GLP-1R), it was previously perceived as an inactive GLP-1 derivative (Deacon, 2004).

Over the past decade, accumulated data have highlighted the cardiovascular effects of 7-36a. However, recent studies demonstrate that 9-36a, once thought to be inactive, has direct effects on the cardiovascular system (Deacon, 2004). A thorough analysis of 7-36a and 9-36a cardiovascular effects, including their commonality, characteristics, and related mechanisms, suggests obvious clinical relevance.

One major focus of this review is to examine cardiovascular effects which differ between 7-36a and its metabolites, 9-36a and 28-36a, and to dissect the interplay of GLP-1R in the heart. This is necessary to better our understanding of GLP-1 biology, and to facilitate our efforts for the development of novel GLP-1 therapies for T2DM and its cardiovascular complications.

## Cardiovascular effects of GLP-1 7-36a

Numerous studies have shown that GLP-1 exerts cardioprotective actions. These include preserving cardiomyocyte and endothelial cell viability *in vitro* (Oeseburg et al., 2010), reducing infarct size and ameliorating cardiac function after myocardial ischemia/reperfusion injury *ex vivo* (Bose et al., 2005), and improving left ventricular function following heart failure in an animal model (Nikolaidis et al., 2004a). In conscious dogs with pacing-induced dilated cardiomyopathy, 7-36a administration significantly increased both basal and insulin-stimulated myocardial glucose uptake, and markedly improved hemodynamics in the absence of increases in plasma insulin (Nikolaidis et al., 2004a). Furthermore, evidence showed that 7-36a infusion improved underlying mitochondrial protein abnormalities and age related accumulation of reactive oxygen species (ROS) in cardiomyocytes of old beagles (Chen et al., 2014).

Consistent with studies in rats which showed that GLP-1 analogs may protect the heart against ischemia/reperfusion injury by improving cardiac energetics and function (Bao et al., 2011), observations from a pilot clinical study suggest that therapies aimed to increase GLP-1 action may counteract oxidative stress, protect from cardiac remodeling, and prevent cardiovascular events in patients with T2DM associated with low circulating levels of GLP-1 (Ravassa et al., 2015). A randomized controlled trial of 172 patients with ST-segment elevation myocardial infarctions also showed a significant reduction in infarct size following treatment with a GLP-1R agonist exenatide (Lønborg et al., 2012).

Due to its short half-life, native GLP-1 needs to be administered using a continuous intravenous infusion to achieve pharmacological effects. Indeed, studies using a continuous intravenous infusion of 7-36a showed beneficial hemodynamic effects in humans with left ventricular systolic dysfunction after acute myocardial infarction (Nikolaidis et al., 2004b). However, since GLP-1 possesses glucose-dependent insulinotrophic effects, it was not clear whether the observed effects were confounded by alterations in insulin and/or glucose concentrations between groups.

To evaluate the effects of GLP-1 on endothelial function independent of insulin and glucose changes, a study on non-diabetic subjects, subjected to euglycemic somatostatin pancreatic clamp during the GLP-1 infusion, suggested that GLP-1 exerts direct beneficial effects on endothelium-dependent vasodilatation in humans (Basu et al., 2007). This notion is further supported by observations from a study in overnight-fasted, healthy young men in which infusion of 7-36a significantly increased microvascular recruitment but not glucose uptake in the skeletal muscle. Remarkably, this effect was also persistently observed in the presence or absence of co-infusion of octreotide, a somatostatin mimetic which attenuates the insulinotropic effects of GLP-1, suggesting that GLP-1-induced vascular effects are independent of insulin action (Sjøberg et al., 2014). Furthermore, it has been shown that, under insulin resistant conditions, insulin action on microvascular recruitment, and glucose transport in skeletal muscles is impaired. GLP-1 treatment improved insulin-mediated microvascular recruitment, muscle glucose uptake, and reversed early stages of insulin resistance induced by high-fat diet feeding in rats (Sjøberg et al., 2015).

Existing evidence regarding GLP-1 effects on cardiac function is limited. One clinical study has shown that 7-36a infusion improves the left ventricular ejection fraction and enhances functional capacity in patients with chronic heart failure (Sokos et al., 2006). Another single-center clinical study also demonstrated that 7-36a treatment was associated with a reduced requirement for pharmacological and mechanical support while achieving comparable hemodynamic outcomes after coronary artery bypass grafting (Müssig et al., 2008). Furthermore, treatment with GLP-1R agonists has consistently demonstrated a reduction in blood pressure in patients with T2DM (Klonoff et al., 2008; Buse et al., 2009). Specifically, it has shown that the long-lasting GLP-1R agonist liraglutide exerted indirect effects on the cardiovascular system through glucose and lipid metabolic control, leading to a decrease in visceral body fat and body weight in obese patients with T2DM (Jendle et al., 2009).

## Protagonist role of both GLP-1 9-36a and 28-36a in cardioprotection

Analysis of the ratio of 7-36a vs. 9-36a in T2DM patients following GLP-1 infusion, with or without concomitant administration of a DPP4 inhibitor, found no evidence that circulating levels of 9-36a were correlated with changes in plasma glucose (Zander et al., 2006). This is in accordance with the fact that, unlike 7-36a, both 9-36a and 28-36a show no effect on insulin secretion or glucose clearance in healthy humans.

The cardioprotective and vasodilatory actions of GLP-1 appear to be mediated through both GLP-1R-dependent and independent pathways (Ban et al., 2008). However, GLP-1 metabolites exert insulinomimetic effects, which may partially account for the cardioprotective effects of GLP-1 (Nikolaidis et al., 2004b, 2005; Ban et al., 2008). This is exemplified by the observations that 9-36a retained vasodilatory and cardioprotective effects in hearts isolated from GLP-1R^−/−^ mice (Ban et al., 2008). Indeed, 9-36a increased myocardial glucose uptake and improved left ventricular performance in dogs with dilated cardiomyopathy (Nikolaidis et al., 2005). In rats, administration of 9-36a after global ischemia significantly improved left ventricular pressure, although its effects in reducing infarct size were marginal (Nikolaidis et al., 2005). In contrast to intact GLP-1, 9-36a directly prevents the production of superoxides induced by high glucose or free fatty acids in human arterial endothelial cells (Ma et al., 2012). Furthermore, 28-36a, another GLP-1 metabolite, attenuates myocardial ischemic injury and significantly reduces infarct size in mice (Mundil et al., 2012).

## Speculation for an alternate GLP-1 receptor

The GLP-1R is a member of the class B1 family of G protein-coupled receptors (Kirkpatrick et al., 2012), mainly expressed in the islet β-cells (Drucker, 2006). Tissue distribution studies of messenger ribonucleic acid encoding GLP-1R suggested its expression in extrapancreatic cell types, including cardiomyocytes and endothelial/vascular smooth muscle cells (Thorens et al., 1993; Bullock et al., 1996). Recent studies showed that the expression of GLP-1R was localized to atrial cardiomyocytes but not in ventricular cardiomyocytes (Kim et al., 2013; Pyke et al., 2014; Richards et al., 2014). The cardioprotective effects of GLP-1 were once thought to be mediated by GLP-1R, given its presence in the cardiovascular system of both human and various animal models (Ravassa et al., 2012), and observations that the mice with a GLP-1R genetic deletion displayed a reduced resting heart rate, elevated left ventricular end-diastolic pressure, increased left ventricular thickness, and impaired contractile responses to insulin and epinephrine (Gros et al., 2003). However, since some of the beneficial cardiovascular effects of 7-36a recently have been shown to be present in mice lacking GLP-1R (Ban et al., 2008), these actions are thus attributed to be 9-36a-mediated through a GLP-1R-independent pathway (Lobb et al., 2012). This perception is further supported by the observations that in GLP-1R^−/−^ mice, both native GLP-1 and 9-36a produced increased coronary flow in isolated hearts and vasodilation of mesenteric arteries in both wild type and GLP-1R^−/−^ mice (Ban et al., 2008). This is consistent with the observations that while the DPP4 resistant GLP-1R agonist exendin-4 is no longer effective in enhancing left ventricular end-diastolic pressure and the rate-pressure product in an isolated rat heart with the simultaneous administration of the GLP-1R antagonist exendin (9-39), the enhancement of the rate-pressure product by 9-36a remains significant (Sonne et al., 2008), suggesting the presence of an unknown GLP-1R which interacts with 9-36a, but not exendin-4 (Tomas and Habener, 2010).

## The overlapping, but distinct functions of 7-36a and its metabolites

**Figure 2:**
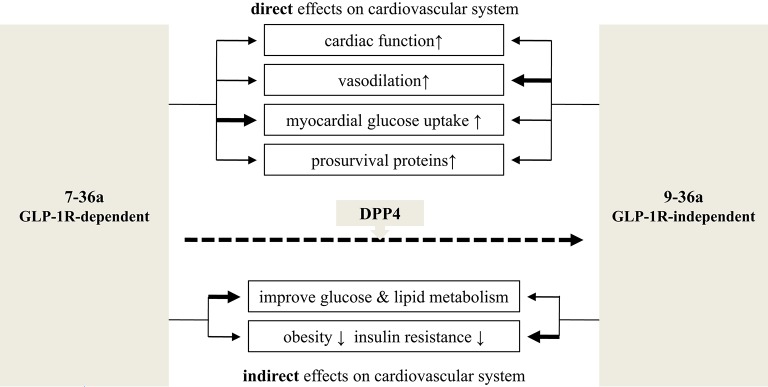
**Cardiovascular effects: 7-36a vs. 9-36a**. The bold line means stronger effects adapted from available experiments. Related references: cardiac function: (Nikolaidis et al., 2004a,b, 2005; Bose et al., 2005; Sokos et al., 2006; Ban et al., 2008; Müssig et al., 2008; Zaruba et al., 2009; Goodwill et al., 2014); vasodilation: (Nikolaidis et al., 2004a; Basu et al., 2007; Ban et al., 2008; Green et al., 2008; Klonoff et al., 2008; Buse et al., 2009; Gardiner et al., 2010; Goodwill et al., 2014); myocardial glucose uptake: (Nikolaidis et al., 2004a, 2005; Ban et al., 2008); prosurvival proteins: (Hausenloy and Yellon, 2007; Sonne et al., 2008; Ban et al., 2010; Chang et al., 2013); improve glucose and lipid metabolism: (Elahi et al., 2008; Klonoff et al., 2008; Jendle et al., 2009); obesity and insulin resistance: (Nikolaidis et al., 2005; Elahi et al., 2008; Jendle et al., 2009; Tomas et al., 2011b).

The rapid degradation of 7-36a to 9-36a by DPP4 makes it hard to determine which form causes which cardioprotective effect. However, by using either GLP-1R or DPP4 knockout mice, some studies have shown overlapping but distinct functions for 7-36a and 9-36a (Figure 2).

**Efficacy comparison** Nikolaidis et al. performed a head-to-head efficacy comparison through a 48 h continuous intravenous infusion of 7-36a and 9-36a in conscious dogs with dilated cardiomyopathy. They found that both peptides increased cardiac output and myocardial glucose uptake without a significant increase in plasma insulin, and improved left ventricular and systemic hemodynamics (Nikolaidis et al., 2005). Therefore, 9-36a mimics the effects of 7-36a in stimulating myocardial glucose uptake and improving left ventricular and systemic hemodynamics. This evidence indicates that 9-36a is an active peptide with comparable effects to 7-36a (Nikolaidis et al., 2005). More evidence substantiates the view that 9-36a is the predominant mediator in conveying GLP-1 action in the cardiovascular system (Ban et al., 2008; Green et al., 2008; Ding et al., 2012). In one study, mass spectrometry was used to analyze coronary effluents of isolated mouse hearts infused with 7-36a or 9-36a. After 30 min of a continuous 7-36a infusion, the vast majority of peptides collected were 9-36a, with only minimal amounts of 7-36a being detected (Ban et al., 2010).

## Mode of action comparison

Studies by Ban et al. showed that while the vasodilatory effects of 7-36a occurred in both wild-type and GLP-1R^−/−^ mice, these effects were abolished by the administration of DPP4 inhibitor sitagliptin and nitric oxide synthase (NOS) blocker L-NNA. They also showed that pretreatment with GLP-1 invoked as much protection from ischemic injury in GLP-1R^−/−^ hearts as in wild type hearts, providing direct genetic evidence for the existence of a GLP-1R–independent pathway coupled to cardioprotection (Ban et al., 2008).

## Range of effect

### Positive inotropy and limiting ischemic injury

An increase in left ventricular diastolic pressure was observed during pretreatment with 7-36a in wild-type but not GLP-1R^−/−^ hearts, whereas pretreatment with 9-36a had no such effect. This suggests that 7-36a, but not 9-36a, has a direct inotropic action via GLP-1R (Wallner et al., 2015). These findings were further supported by observations of reduced myocardial infarction size and improved survival of DPP4 knockout mice (Sauve et al., 2010). A study by Goodwill et al. was conducted in lean, open chest and anesthetized swine which received systemic infusions of saline or GLP-1 (7-36a or 9-36a), followed with coronary ligation to induce regional ischemia, showing that unlike 9-36a, treatment with 7-36a significantly improved cardiac output during regional myocardial ischemia by increasing ventricular preload without changes in cardiac inotropy (Goodwill et al., 2014).

In another study by Ban et al. effects of 7-36a and 9-36a on isolated mouse hearts undergoing ischemia-reperfusion injury were studied (Ban et al., 2008). They demonstrated that pretreatment with 7-36a but not 9-36a, and post-treatment with 9-36a resulted in significant functional recovery (indexed by left ventricular developed pressure) from the ischemia-reperfusion injury in both wild-type and GLP-1R^−/−^ hearts compared with untreated controls.

Collectively, these findings suggest that the cardiovascular effects of 7-36a include inotropic action, glucose uptake, and ischemic preconditioning dependent on GLP-1R. In contrast, 9-36a affects post-ischemic recovery of cardiac function independently of GLP-1R.

### Vasodilation

It is important to note that unlike 7-36a or 9-36a, exendin-4 did not produce vasodilatation or cyclic guanosine monophosphate (cGMP) release in mesenteric arteries from both wild-type and GLP-1R^−/−^ mice that were partially preconstricted with phenylephrine, and the vasodilatory responses from both 7-36a and 9-36a in arteries were completely abolished by pretreatment with the NOS blocker L-NNA (Ban et al., 2008); it is suggested that the vascular effects of GLP-1 are mediated via 9-36a and a GLP-1R–independent mechanism acting partially through NOS-dependent cGMP formation.

To determine whether the conversion of 7-36a to 9-36a is required for the vasodilatory action of GLP-1, Ban et al. conducted a study using DPP4 inhibitor sitagliptin in both wild-type and GLP-1R^−/−^ mice. Remarkably, DPP4 inhibition to prevent generation of 9-36a attenuated, but did not abolish, the beneficial effects of 7-36a on vasodilation, suggesting that both native GLP-1 (7-36a) and its metabolite 9-36a possess vasodilatory action. Since both 7-36a and 9-36a persistently induced vasodilation in arteries from GLP-1R^−/−^ mice (Ban et al., 2008), these data strongly support the existence of a vasodilatory signaling mechanism for 7-36a and 9-36a that is independent of the known functional GLP-1R.

The effects of 9-36a on endothelial nitric oxide synthase (eNOS) have been examined in human umbilical vein endothelial cells (HUVECs). The results revealed that 9-36a increased NO release, eNOS activity and expression in HUVECs, suggesting that the vasodilatory effect of NO is a potential mechanism underlying the protective effects of 9-36a on the cardiovascular system (Ding and Zhang, 2012). A recent study showed that the administration of 7-36a in both physiological and supraphysiological concentrations to a femoral artery infusion increased microvascular recruitment, but not glucose uptake in human and rat skeletal muscle (Sjøberg et al., 2014), suggesting that the vascular effects of GLP-1 are independent of insulin action. Additionally, GLP-1 induced vasodilation of isolated pre-constricted mesenteric arteries *ex vivo* in a NOS-dependent manner in the absence of insulin (Ban et al., 2008).

Meta-analysis of clinical trials showed that blood pressure reduction is frequently observed with chronic administration of GLP-1R agonists in patients with T2DM (Wang et al., 2013; Sun et al., 2015). However, administration of DPP-4 inhibitors showed modest reduction or even no changes in blood pressure in patients with T2DM and in apolipoprotein E-deficient (ApoE^−/−^) mice which were susceptible to atherosclerosis (Matsubara et al., 2012; Zhang and Zhao, 2016). These findings highlight the significance of the GLP-1 metabolite 9-36a in mediating the cardioprotective and vasodilatory actions of GLP-1 itself. Nevertheless, clinical studies specifically designed to evaluate the potential cardiovascular benefits of 9-36a in patients with T2DM are warranted.

### Cytoprotection against oxidative stress

Picatoste et al. reported that native GLP-1 and 9-36a induced anti-apoptotic/necrotic, anti-hypertrophic, and anti-fibrotic actions on high fat and/or high glucose-stimulated cardiac cells *in vitro*. They observed that while cardiac fibroblasts did not express GLP-1R, both native GLP-1 and 9-36a reduced pro-fibrotic expression after stimulation with high concentrations of palmitate or glucose (Picatoste et al., 2013). The anti-fibrotic effect of GLP-1 is reversed by sitagliptin pretreatment, suggesting that 9-36a plays a direct role. In addition, 9-36a was observed to exert anti-oxidant effects on cardiac and vascular cells (Picatoste et al., 2013).

### Mitigation of inflammation, atherosclerosis and stent restenosis

Liraglutide, a GLP-1R agonist, was observed to reduce tumor necrosis factor alpha induced nuclear factor KB activation and lower production of inflammatory genes in HUVECs and murine endothelial cell line SVEC4, partly via AMP-activated protein kinase (AMPK) activation (Hattori et al., 2010). Another GLP-1 receptor agonist, exendin-4, was reported to reduce the accumulation of monocytes/macrophages in the arterial wall of ApoE^−/−^ mice partly through activation of the cAMP/PKA pathway, which may contribute to attenuation of atherosclerosis (Arakawa et al., 2010). Without affecting glucose metabolism, exendin-4 consistently reduced the proliferation of smooth muscle cells and attenuated neointimal formation after vascular injury in normal C57BL/6 mice, which may potentially be suitable for the prevention of atherosclerosis and post stent restenosis (Goto et al., 2011; Hirata et al., 2013).

Sitagliptin, a DPP4 inhibitor, reduced plaque inflammation and increased plaque stability, potentially through inhibition of monocyte migration and macrophage metalloproteinase-9 release in ApoE^−/−^ mice (Vittone et al., 2012). However, in the PROLOGUE study, no evidence was provided during the 24-month follow-up period that sitagliptin in addition to conventional therapy could slow the progression of carotid intima-media thickening, a surrogate marker for evaluating atherosclerotic cardiovascular disease, in patients with T2DM compared with conventional therapy alone (Oyama et al., 2016). The cause of this discrepancy between basic experiments and clinical trials remains unknown. Since, according to the UKPDS and VADT studies efficacy against cardiovascular events may be demonstrated over several years or even longer, it cannot be excluded that the 24 month follow-up period in the PROLOGUE study may be too short to evaluate the anti-atherosclerotic effects of sitagliptin.

To determine whether the effects of GLP-1 on atherosclerosis are mediated through a GLP-1R dependent pathway or not, 7-36a, 9-36a, and 28-36a were overexpressed for 12 weeks in ApoE^−/−^ mice on a high-fat diet using an adeno-associated viral vector system. Compared to LacZ (control), all three GLP-1 constructs reduced plaque macrophage infiltration as well as plaque metalloproteinase-9 expression, and stabilized plaque by increasing plaque collagen content and fibrous cap thickness in the aorta (Burgmaier et al., 2013). The fact that liraglutide, exendin-4, sitagliptin, and native GLP-1, as well as its metabolites, had comparable anti-atherosclerotic effects, implying that this cardiovascular benefit is mediated through both GLP-1R-dependent and independent pathways.

## Enigma of GLP-1 receptor in cardiovascular system

### GLP-1 signaling in cardiomyocytes

The signaling mechanism underlying GLP-1 actions in the islet β-cells has been extensively studied. Activation of the β-cell GLP-1R, a G-protein coupled receptor, stimulates the cyclic adenosine monophosphate (cAMP) dependent signaling that leads to insulin secretion, and phosphoinositide 3-kinase (PI3K)/protein kinase B (Akt) signaling pathways which promote β-cell growth and survival (Wang and Brubaker, 2002; Wang et al., 2004). To investigate the relevant molecular mechanisms underlying GLP-1R–dependent cardioprotection, Drucker and colleagues assessed GLP-1 induced cytoprotective pathways in normal and diabetic mice with experimental myocardial infarction (Noyan-Ashraf et al., 2009). They found that the pretreatment of GLP-1R agonist liraglutide significantly increased the survival rate due to reduced cardiac rupture and infarct size and improved cardiac output. This was associated with increased expression and activity of cardioprotective genes in the mouse heart, including prosurvival kinase Akt and GSK3β, the nuclear receptor PPARβ/δ, and the redox-sensitive basic leucine zipper transcription factor Nrf2, and concomitantly, decreased expression of pro-apoptotic gene caspase-3 in a GLP-1R antagonist exendin (9-39) sensitive fashion. These findings suggest that GLP-1 activates pro-survival signaling pathways in the normal and diabetic mouse heart, leading to improved cardiac outcomes and enhanced survival after myocardial infarction in mice (Noyan-Ashraf et al., 2009). It is known that activation of apoptosis contributes to cardiomyocyte dysfunction. Using *in vitro* culture system in murine HL-1 cardiomyocytes, Ravassa et al. evaluated cardiac cytoprotective signaling of native GLP-1. They found that GLP-1 treatment protected the cardiomyocytes from apoptosis induced by the protein kinase inhibitor staurosporine and saturated fatty acid palmitate, in which the protective effects were attenuated by PI3K and/or ERK1/2 inhibition, suggesting a role for PI3K and ERK1/2 in mediating GLP-1 cytoprotective effects in the cardiomyocytes (Ravassa et al., 2011).

Notably, effects of GLP-1 on myocyte electrophysiology were examined. Studies using whole-cell patch clamp on isolated canine left ventricular myocytes showed that extracellular perfusion of 7-36a increased cardiac voltage-gated L-type Ca^2+^ current, which was blocked by pre-treatment of GLP-1R antagonist exendin (9-39), or intracellular dialysis with a protein kinase A inhibitor. It is presumed that, via a GLP-1R and cAMP dependent signaling pathway, GLP-1 increases these Ca^2+^ channels activities, leading to Ca^2+^ influx and increases in the contractility of cardiac myocytes (Xiao et al., 2011).

To address whether 9-36a induced cardioprotective actions are coupled to classical GLP-1R, Husain and colleagues compared cardiac protective effects induced by 9-37a and GLP-1R agonist exendin-4 in the wild-type and GLP-1R^−/−^ mice in the presence or absence of exendin (9-39). They demonstrated that after ischemia-reperfusion (I/R) injury of isolated mouse hearts, both 9-36a and exendin-4 improved functional recovery and reduced infarct size. In cultured neonatal mouse cardiomyocytes, both peptides increased levels of cAMP and phosphorylation of ERK1/2 and PI3K/Akt activation. Furthermore, both peptides improved mouse cardiomyocyte viability and reduced lactate dehydrogenase release and caspase-3 activation (Ban et al., 2010). These data provide evidence that both 9-36a and exendin-4 exert cytoprotective effects through PI3K/Akt and ERK1/2 dependent signaling pathways. Furthermore, unlike exendin-4, the cardioprotective effects of 9-36a were preserved in GLP-1R^−/−^ cardiomyocytes, suggesting that 9-36a induced cardioprotective effects via an exendin (9-39)-sensitive but distinct from the classical GLP-1R.

### GLP-1 signaling in endothelial cells

Studies by Hattori et al. showed that the administration of liraglutide to HUVECs dose-dependently enhanced eNOS phosphorylation, increased NO production, and suppressed NF-kB activation (Hattori et al., 2010). However, these effects are attenuated by the AMPK inhibitor compound C or AMPK small interfering RNA, suggesting that these favorable actions of liraglutide are mediated at least in part through AMPK activation. This observation is corroborated by further evidence showing that exendin-4, 7-36a, and 9-36a all stimulates proliferation of human coronary artery endothelial cells (HCAECs) through eNOS and Akt activation (Erdogdu et al., 2010), implying that involvement of both GLP-1R-dependent and independent pathways.

Interestingly, in a single-blind random crossover study, subjects underwent intravenous infusion of human recombinant GLP-1 for 105 min and had their endothelial function and insulin sensitivity measured with ultrasonography through flow-mediated vasodilation (FMD) and hyperinsulinemic isoglycemic clamp technique, respectively; 7-36a increased the FMD response and relatively (16%), albeit not significantly, affected insulin sensitivity in T2DM subjects, without any effects on FMD and insulin sensitivity in healthy subjects (Nyström et al., 2004). This seems inconsistent with previous studies, which demonstrated an improvement in insulin sensitivity with a 6 week course of 7-36a in patients with T2DM (Zander et al., 2002), however, it cannot be excluded that during this very short time (105 min) GLP-1 may not affect insulin sensitivity, and the beneficial endothelial effects of GLP-1 in patients with T2DM were not fully dependent on improvements in insulin sensitivity.

To evaluate the effects of exendin-4 and 7-36a on lipoapoptosis and underlying mechanisms, incubation with exendin-4 and 7-36a protected HCAECs against lipoapoptosis; this effect was attenuated by PKA, PI3K/Akt, eNOS, and p38 MAPK, as well as JNK inhibitors and GLP-1R antagonists; the fact that 9-36a failed to reduce apoptosis in HCAECs also favors the view that this anti-lipoapoptotic effect is mediated through a GLP-1R dependent pathway (Erdogdu et al., 2013).

### GLP-1 signaling in vascular smooth muscle cells

GLP-1R has been identified in the vascular smooth muscle cells (VSMCs) of both rodents and humans (Pyke et al., 2014; Richards et al., 2014). VSMCs play an important role in the initiation and progression of atherosclerosis (Doran et al., 2008). It has shown that treatment with the critical atherogenesis factor oxidized low-density lipoprotein (ox-LDL), downregulated GLP-1R expression, and was also subsequently attenuated by lectin-like oxidized low-density lipoprotein scavenger receptor-1 (LOX-1) antibody in human VSMCs. Conversely, over-expression of LOX-1 decreased the inhibitory effect of liraglutide on the mitochondrial ROS production in human VSMCs (Dai et al., 2013). Collectively, these results indicate that Liraglutide reduced ox-LDL-induced mitochondrial ROS generation in human aortic smooth muscle cells, furthermore demonstrating that LOX-1 may play a connective role between GLP-1 activation and ROS interaction (Dai et al., 2013).

Consistent with previous studies on smooth muscle cells and wire-mediated endovascular injury in C57BL/6 mice administered with exendin-4 over 4 weeks (Hirata et al., 2013), in SD rats subjected to balloon injury of carotid artery, 4 weeks of exendin-4 treatment selectively reduced smooth muscle cell proliferation and increased smooth muscle cell apoptosis (Eriksson et al., 2015). This proapoptotic effect in smooth muscle cell seems to be mediated through a GLP-1R dependent pathway, which can be prevented by the GLP-1R antagonist exendin (9-39).

## GLP-1 cleavage by NEP

Neutral endopeptidase (NEP) 24.11 is an endopeptidase expressed in endothelial cells and VSMCs (Graf et al., 1995; Hupe-Sodmann et al., 1995). It cleaves GLP-1 at several sites into various metabolites including 28-36a and 32-36a (Hupe-Sodmann et al., 1995; Plamboeck et al., 2005) (Figure 1). NEP inhibition, which enhances cGMP, has been proposed to modulate the progression of heart failure (Birner et al., 2012) and the prevention of atherogenesis (Ichiki et al., 2013). A number of studies suggested that the downstream metabolites of 7-36a and 9-36a under cleavage by NEP 24.11 may account for insulinomimetic effects of 7-36 independently from GLP-1R (Tomas et al., 2011a; Sharma et al., 2013). Beneficial cardiac effects have been traced to 28-36a, while 32-36a seems to confer an increase in fatty acid oxidation and helps to control weight gain (Tomas et al., 2011a, 2015).

## 9-36a is effective in insulin-resistant-associated cardiovascular diseases

A study by Gardiner et al. evaluated the regional hemodynamic effects of 7-36a and 9-36a under physiological conditions through a persistent infusion of the two peptides in conscious and chronically instrumented rats (Gardiner et al., 2010). They found that 7-36a exerted obvious regional hemodynamic effects including tachycardia, a rise in blood pressure, renal and mesenteric vasoconstriction, and vasodilation in the hindquarters. 9-36a, however, was devoid of any cardiovascular actions in this study (Gardiner et al., 2010). This finding seemingly contrasts with previous studies showing that 9-36a improves left ventricular performance and systemic hemodynamics in conscious dogs with dilated cardiomyopathy and myocardial insulin resistance (Nikolaidis et al., 2005). It is possible that, *in vivo*, the cardiovascular actions of 9-36a only become apparent under certain pathophysiological conditions, such as insulin-resistant states. This explanation is also supported by another study which demonstrated that 9-36a suppresses hepatic glucose production when infused into obese and insulin resistant human subjects, but not in healthy human subjects (Elahi et al., 2008). Furthermore, 9-36a treatment attenuated the insulin resistance and obesity induced by a high-fat diet feeding in mice (Tomas et al., 2011b). Since cardiometabolic responses to native GLP-1 were impaired in obesity and T2DM (Moberly et al., 2013), these studies support that 9-36a may be particularly suitable for treating cardiovascular diseases co-morbid with insulin-resistant-associated diseases such as obesity, T2DM and metabolic syndrome (Ban et al., 2008; Sonne et al., 2008).

## DPP4 inhibitors may impede GLP-1 insulinomimetic effects

### DPP4 cleavage of substrates other than GLP-1, and its potential outcome

DPP4 inhibition may also prolong the action of other peptide hormones cleaved by the protease including gastric inhibitory polypeptide (GIP), substance P, B-type natriuretic peptide, and pituitary adenylate cyclase-activating peptide (Mentlein, 2004; Brandt et al., 2006; Sauve et al., 2010). It has been postulated that the stabilization of these peptide hormones, neuropeptides, and chemokines may enhance inflammatory or allergic reactions (Mest and Mentlein, 2005), increase the risk of tumors (Masur et al., 2006; Kajiyama et al., 2010), and may harm the antithrombogenic nature of the endothelium (Krijnen et al., 2012). Moreover, despite limited data on its cardiovascular relevance, GIP receptors have been recognized in the atrial and ventricular tissue of rodents, and activation of these receptors has been linked to lipogenesity (Usdin et al., 1993; Hansotia et al., 2007; Kim et al., 2007). Whether or not the increase in GIP due to DPP4 inhibition in humans is responsible for some of cardiovascular effects of DPP4 inhibitors, and if this is clinically relevant, has yet to be determined.

### Preclinical studies, cardioprotection as a result of DPP4 inhibition

A recent study is suggestive of cardiac fibrosis and impairment of cardiac function following treatment with a DPP4 inhibitor in diabetic mice (Mulvihill et al., 2016). However, the majority of preclinical studies allude to cardioprotection as a result of DPP4 inhibition. It was shown that the treatment with DPP4 inhibitor sitagliptin improved cardiovascular outcomes after myocardial infarction in non-diabetic mice; similar results were also obtained in non-diabetic DPP4^−/−^ mice (Sauve et al., 2010). As stromal cell-derived factor-1 (SDF-1) is an endogenous DPP4 substrate which can mobilize endothelial progenitor cells to sites of vascular or myocardial injury, it is intriguing that DPP4 inhibition enhances SDF-1, improves cardiac function and survival after acute myocardial infarction in mice, and increases circulating endothelial progenitor cells in patients with T2DM (Zaruba et al., 2009; Fadini et al., 2010).

### Clinical studies, DPP4 inhibitors vs. GLP-1 analogs/GLP-1R agonists

There are two major classes of GLP-1 based therapies that have been clinically used. One class is the DPP4 inhibitors, such as sitagliptin, vildagliptin and saxagliptin. The other class is the GLP-1 analogues or GLP-1R agonists (e.g., liraglutide and exenatide). There are some important differences between the two classes. There are a number of potential detrimental effects associated with the DPP4 inhibition, along with the inferior clinical efficacy of DPP4 inhibitors in reducing HbA1c and obesity compared with GLP-1 analogs/GLP-1R agonists (Association, 2016). Furthermore, the changes in circulating levels of “active GLP-1” are markedly different between the two approaches, with DPP4 inhibitors generally raising concentrations of endogenous 7-36a by 2- to 4-fold whilst pharmaceutical circulating levels of GLP-1R agonists can be 10-fold greater than endogenous levels (Pabreja et al., 2014). The LEADER study, a large double-blind, randomized controlled trial, has demonstrated that the rate of the first occurrence of death from cardiovascular causes, non-fatal myocardial infarction, or non-fatal stroke among patients with T2DM is lower with liraglutide than with a placebo (Marso et al., 2016a). In the SUSTAIN-6 study, semaglutide, another GLP-1 analog, was associated with a significantly lower risk of the primary composite outcome of death from cardiovascular causes, non-fatal myocardial infarctions, or non-fatal strokes than did those receiving a placebo (Marso et al., 2016b). However, the SAVOR-TIMI 53 trial (saxagliptin) (Scirica et al., 2013), EXAMINE trial (alogliptin) (White et al., 2013), and TECOS trial (sitagliptin) (Green et al., 2015) all failed to improve cardiovascular outcomes with DPP4 inhibition compared to the placebo therapy. The rate of hospitalization for heart failure was found to be increased with saxagliptin (Scirica et al., 2013). The greater beneficial cardiovascular effects and superior clinical efficacy in the glycemic control and improving obesity achieved by therapies using GLP-1 analogs/GLP-1R agonists than those by DPP4 inhibition, presumably due to relatively and pharmacologically higher efficacy in the activation of the GLP-1R in these subjects on GLP-1 analogs/GLP-1R agonist therapies. Nevertheless, direct evidence is required to address whether or not the shortage of 9-36a caused by DPP4 inhibitors may be responsible for these differences.

## Current GLP-1 treatment strategies and their inadequacy

Numerous studies showed that DPP4 cleavage initiates degradation pathways that lead to the inactivation and disposal of GLP-1 (Deacon, 2004; Plamboeck et al., 2005), current GLP-1 therapies, either under clinical investigation or approved, were thus developed with a goal of avoiding GLP-1 degradation by DPP4 (Drucker, 2006). The two main strategies of GLP-1 therapy—the use of a DPP4-resistant GLP-1 analogs/GLP-1R agonists, and the use of DPP4 inhibitors—have been developed and clinically used for treating diabetes with the aim of prolonging the half-life of 7-36a.

Recent findings suggest that the cardioprotective effects of DPP4 inhibitors and DPP4-resistant GLP-1 analogs/GLP-1R agonists are limited compared to those of GLP-1 in its native form. In cultured neonatal mouse cardiomyocytes exposed to either hypoxia reoxygenation or H_2_O_2_, both 9-36a and exendin-4 can improve cardiomyocyte viability and reduce lactate dehydrogenase release and caspase-3 activation, by increasing levels of cAMP and prosurvival proteins including phosphorylated ERK1/2 and Akt (Ban et al., 2010; Chang et al., 2013). The beneficial effects of both peptides were diminished when co-incubated with the GLP-1R antagonist Exendin (9-39). However, the cAMP stimulation and prosurvival effects of 9-36a, but not exendin-4, remained evident in GLP-1R^−/−^ cardiomyocytes, suggesting the interaction of 9-36a with a presumably alternative receptor (Ban et al., 2010). In addition, 9-36a, but not exendin-4, improved the survival of human aortic endothelial cells after exposure to hypoxia reoxygenation, or H_2_O_2_, through a NOS-dependent mechanism (Ban et al., 2010). *Ex vivo* studies using rat conduit arteries established a vasorelaxation response for both 7-36a and 9-36a, while exendin-4 produced no vasodilatory activity (Nathanson et al., 2009). Notably, strategies utilizing DPP4-resistant GLP-1 analogs or GLP-1R agonists and DPP4 inhibition could not fully invoke the cardiovascular effects of native GLP-1.

## Increased endogenous GLP-1 production and cardiovascular benefits

Bariatric surgery is an effective treatment for patients with severe obesity. It results in sustained weight loss, remarkable improvement of T2DM, and significant reduction of cardiovascular events (Sjöström et al., 2012; Cotugno et al., 2015). Notably, in a retrospective study conducted in patients with T2DM and severe obesity, bariatric surgery was more effective in reducing cardiovascular risk profiles than standard hypoglycemic therapy in conjunction with liraglutide (Cotugno et al., 2015). Native GLP-1 secretion in response to meals is dramatically increased after bariatric surgery, particularly after Roux-en-Y gastric bypasses and sleeve gastrectomies. Although reduced fasting DPP4 activity has been observed after the Roux-en-Y gastric bypass surgery, the precise mechanisms have not yet been fully elucidated. It has shown that postoperative improvement of glucose tolerance as well as insulin secretion is reduced by the GLP-1 receptor antagonist, exendin (9-39) (Karra et al., 2010; Alam et al., 2011; Manning et al., 2015). This evidence suggests that increased endogenous GLP-1 production is responsible, in part, for these beneficial effects.

An alternative strategy for enhancing endogenous GLP-1 secretion may be to increase the growth and viability of L cells. Recent studies suggested that lipotoxicity occurring in the GLP-1 secreting L cells could represent a mechanism underlying impaired GLP-1 secretion in response to meal ingestion in T2DM patients. Indeed, *in vitro* studies showed that treatment of L cells with metformin, insulin, or exendin-4 could protect against L cell lipoapotosis, and stimulate GLP-1 secretion from the L cells (Kappe et al., 2012, 2013a,b; Kuhre et al., 2016). In an *in vivo* study in mice received 12-weeks control or high-fat diet, a 55% but no significant (*p* = 0.134) reduction of the number of GLP-1-positive cells was indicated in the high-fat diet group, and this effect was indicated to be reversed by 14 days administration of metformin; however, no significant effect can be determined of a high-fat diet or metformin treatment on fasting or prandial serum GLP-1 (7-36a and 9-36a) levels (Kappe et al., 2014). Therefore, further studies are necessary to confirm the treatment effect of metformin on L cells *in vivo*.

Increasing evidence demonstrates that both 7-36a and 9-36a exert cardiovascular effects. It is conceivable that a therapeutic strategy to prolong native GLP-1 action (Wang et al., 2010) may have superior beneficial cardiovascular effects along with glycemic and metabolic control.

## Conclusions

Recent advances in GLP-1 biology and incretin-based therapies suggest that the mechanism of action for native GLP-1 appears to be a varied spectrum characterized by insulinotropic and insulinomimetic features. Compelling evidence suggests that 7-36a and 9-36a have direct cardiovascular effects which are overlapping in some ways, but are distinct and complementary in others. While 7-36a exerts cardiovascular effects in a GLP-1R-dependent fashion, mainly improving myocardial glucose uptake, 9-36a acts through GLP-1R-independent processes, largely accounting for vasodilation, amelioration of endothelial dysfunction, increased cardiac function, and cytoprotection against ischemic-reperfusion injury. As such, it may be particularly relevant with regards to insulin-resistance-associated cardiovascular diseases (Nikolaidis et al., 2005; Ban et al., 2008; Erdogdu et al., 2010). Furthermore, both 7-36a and 9-36a exert indirect effects on the cardiovascular system, such as improving metabolic control to make the prognosis of cardiovascular diseases more optimistic. Additionally, the cleavage of native GLP-1 (7-36) by alternative pathways such as NEP 24.11 into metabolites may account for some of the cardiovascular benefits of native GLP-1 either directly or indirectly, by correcting metabolic syndrome (Tomas et al., 2011c, 2015).

Therefore, the two current GLP-1-therapy strategies, DPP4-resistant GLP-1 analogs/GLP-1R agonists and DPP4 inhibitors, may not have an optimistic outlook due to the lack of 9-36a and its associated cardioprotective virtues, as well as previously mentioned detrimental effects. The development of new strategies in GLP-1-therapy to simultaneously prolong the actions of native GLP-1 and its metabolites may achieve profound therapeutic effects in the context of glycemic control and cardiac protection.

## Author contributions

QW contributed to the conception and design of the study; JL and JZ contributed to the literature search; JL, JZ, and QW contributed to the data analysis; JL, JZ, SW, HL, AF, and QW contributed to the discussion; JL and QW contributed to drafting the article; SW, HL, and AF contributed to the editorial reading; HL and AF contributed to the reference accuracy; QW is responsible for the integrity of the work as a whole. All authors have revised the manuscript critically for important intellectual content and given final approval of the version to be published.

## Funding

This work was supported by grants from the Canadian Diabetes Association (CDA, OG-3-13-4066-QW), Juvenile Diabetes Research Foundation (JDRF, 2015-64-Q-R), and National Science Foundation China (NSFC, 81570518, 81370877).

### Conflict of interest statement

The authors declare that the research was conducted in the absence of any commercial or financial relationships that could be construed as a potential conflict of interest.

## Abbreviations

T2DM: type 2 diabetes mellitus
GLP-1: Glucagon-like peptide 1
DPP4: diaminopeptidyl peptidase-4
7-36a: GLP-1 (7-36) amide
9-36a: GLP-1 (9-36) amide
28-36a: GLP-1 (28-36) amide
32-36a: GLP-1 (32-36) amide
GLP-1R: GLP-1 receptor
ROS: reactive oxygen species
NO: nitric oxide
NOS: nitric oxide synthase
eNOS: endothelial nitric oxide synthase
cGMP: cyclic guanosine monophosphate
cAMP: cyclic adenosine monophosphate
HUVECs: human umbilical vein endothelial cells
HCAECs: human coronary artery endothelial cells
PI3K: phosphoinositide 3-kinase
Akt: protein kinase B
ERK1/2: extracellular signal-regulated kinases 1 and 2
NEP: Neutral endopeptidase
GIP: gastric inhibitory polypeptide
SDF-1: stromal cell-derived factor-1
AMPK: AMP-activated protein kinase
VSMCs: vascular smooth muscle cells
ox-LDL: oxidized low-density lipoprotein
LOX-1: lectin-like oxidized low-density lipoprotein scavenger receptor-1
FMD: flow-mediated vasodilation.
